# Neurosurgery at Baylor Scott & White Health: Recent History and a Decade of Neurosurgery Residency Training

**DOI:** 10.7759/cureus.53181

**Published:** 2024-01-29

**Authors:** Samuel R Daly, Anthony V Nguyen, Laura K Reed, Ibrahim M Elnihum, Frank S Harris, David Garrett, Erxi Wu, James K Cooper, Awais Z Vance, Jason H Huang

**Affiliations:** 1 Neurosurgery, Baylor Scott & White Medical Center - Temple, Temple, USA

**Keywords:** baylor scott and white health, future directions, history of surgery, residency program, neurosurgery residency

## Abstract

Neurosurgery at Baylor Scott & White Memorial Hospital in Temple, Texas began as a division in the Department of Surgery many decades ago. The hospital has long served as the flagship tertiary referral center for the Baylor Scott & White healthcare system, which merged in 2013 with Baylor University Medical Center, a hospital system based in Dallas. It is now the largest non-profit hospital system as well as the most awarded hospital system by the US News and World Report within the state of Texas. The Department of Neurosurgery was established at Baylor Scott & White Memorial Hospital in the 2006-2007 academic year. Between then and 2014, four neurosurgeons served as department chair or interim chair: Dr. Robert Buchanan, Dr. Gerhard Friehs, Dr. Ibrahim El Nihum, and Dr. David Garrett Jr. In 2014, Dr. Jason Huang was appointed chairman after a national search and established the neurosurgery residency program in 2015. The department has undergone tremendous growth under the leadership of Dr. Huang, and the residency program is a priority of the department. Surgical excellence is honed at primarily three campuses: Baylor Scott & White Memorial Hospital, Baylor Scott & White McLane Children's Medical Center, and Baylor Scott & White Medical Center - Hillcrest. In this editorial, we provide a brief history of the institution, a recent history of the neurosurgical presence at Baylor Scott & White Memorial Hospital in Temple, Texas, and briefly describe the program’s future directions under the continued leadership of Dr. Jason Huang.

## Editorial

Brief history of Baylor Scott & White Health

**Figure 1:**
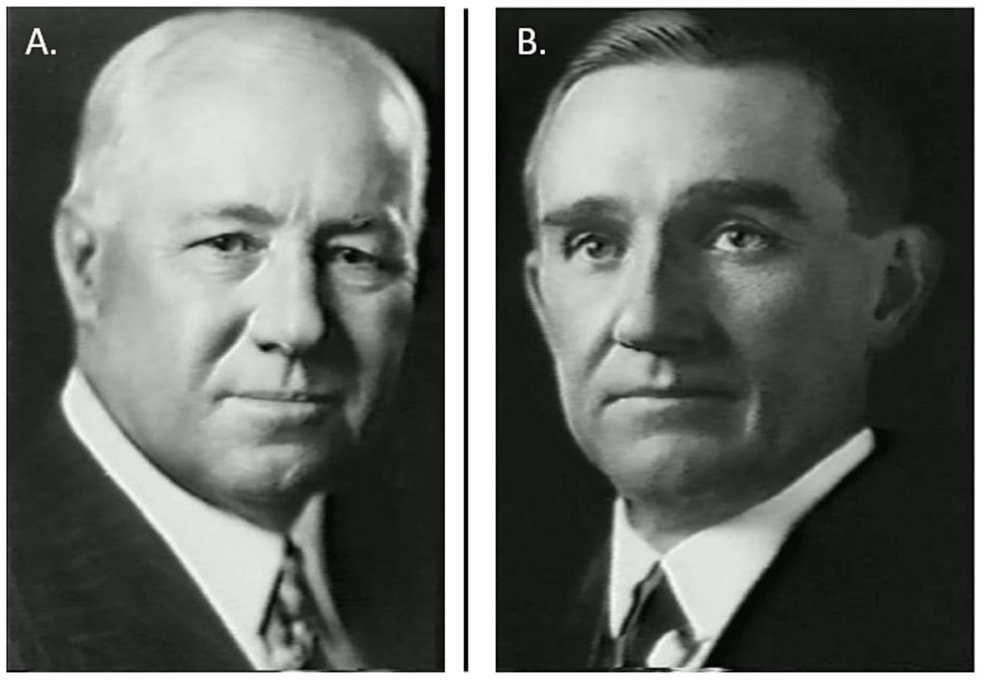
Founders of Scott and White Medical Center Arthur C. Scott, MD (A) and Raleigh White II, MD (B), the founders of Scott and White Medical Center. (Images were adapted from the Baylor Scott & White Health website, and permission has been granted to use them in this publication.)

Baylor Scott & White Health in Temple, Texas has its roots in the Santa Fe Railroad Hospital - a small hospital established in 1891 to serve the town built around the railroad yard. Arthur C. Scott, MD moved to Temple, TX in 1892 to become the Chief Surgeon (Figure 1A), and he quickly built a busy practice. In 1895, Dr. Scott held a competitive examination to fill the vacant position of House Surgeon, and the highest score was achieved by Raleigh White II, MD (Figure 1B). Dr. Scott and Dr. White signed a full partnership agreement in 1897, and they founded the Temple Sanitarium in 1904, which was renamed Scott and White Hospital in 1922. Over the next century, the hospital grew significantly, and multiple residency training programs were established, including one in General Surgery in the 1920’s. Scott and White became affiliated with Texas A&M Medical School in 1979 and began training medical students. In 2013, Scott and White Hospital merged with Baylor University Medical Center to form Baylor Scott & White Health.

**Figure 2:**
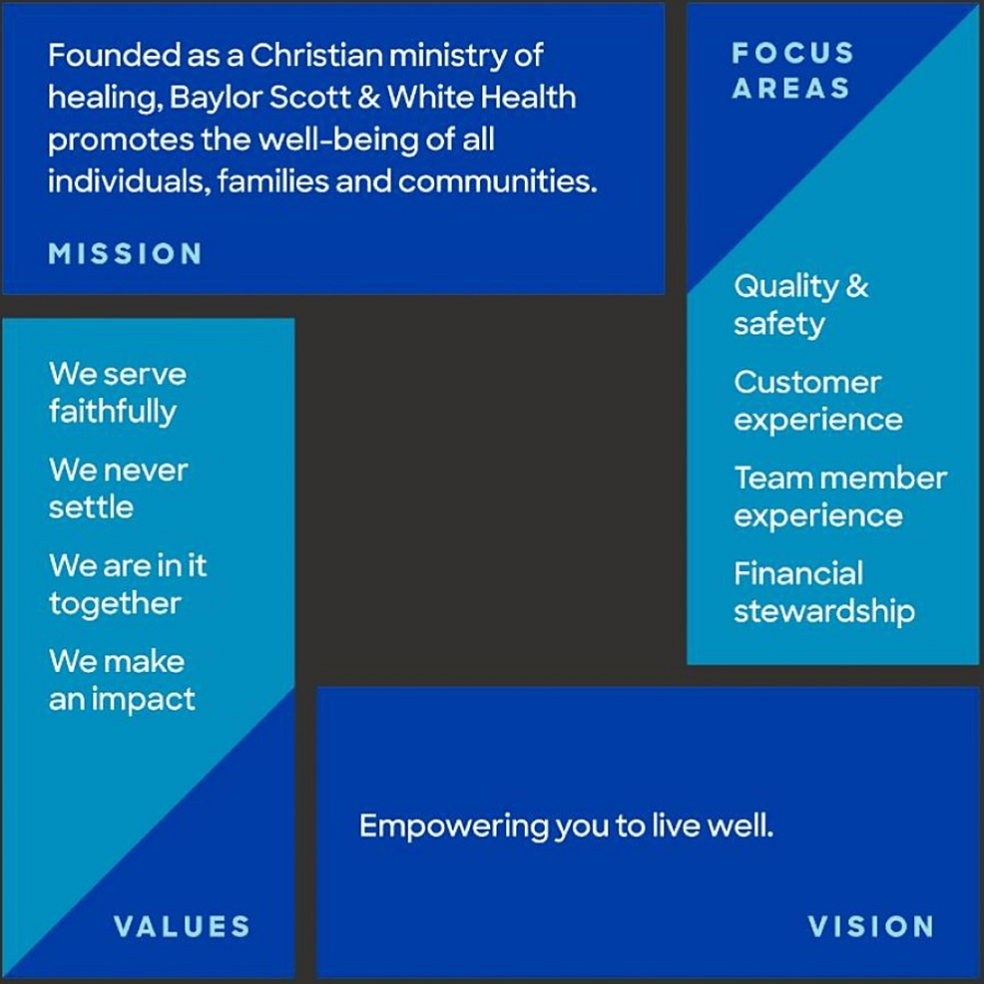
The CORE of Baylor Scott & White Health Image was adapted from the Baylor Scott & White Health website, and permission has been granted to use it in this publication.

Baylor Scott & White Memorial Hospital functions as the flagship tertiary referral center within Baylor Scott and White Health, which is currently the largest not-for-profit healthcare system in Texas. It is also the most awarded healthcare system in the state of Texas by the US News and World Report, and in 2023, the Temple campus achieved the top overall rank in Fortune's "15 Top Major Teaching Hospitals" in the United States [1]. There are currently 19 residency training programs and 24 fellowship programs with more than 400 residents and fellows. The institution's mission statement, values, and ambition set high standards to continue to provide state-of-the-art care to all patients (Figure 2).

History of the Department of Neurosurgery

The Division of Neurosurgery existed within the Department of Surgery for many decades. In the 1970s, Dr. Robert H. Wilkins, who later became Chair of Neurosurgery at Duke University School of Medicine, served as Chief of the Division of Neurosurgery at Scott and White Clinic for two years (1973-1975) [2]. In 1992, Ibraham El Nihum, MD joined the department and became the Director of the Division of Neurosurgery. Dr. El Nihum attended medical school at the University of Benghazi in Libya, and he completed his residency at Victoria General Hospital in Vancouver, Canada (University of British Columbia). Prior to joining the faculty at Scott and White Hospital, he did two fellowships at the University of California San Diego and one at the University of Texas - Southwestern, where he was the first pediatric neurosurgery fellow. Dr. El Nihum was joined on the faculty by Deborah Henry, MD in 1993, Frank Harris, MD in 1994, and William White, MD in 1996.

When Dr. Harris started his career at Scott and White Hospital in 1994, he became the Director of the Division of Neurosurgery. Dr. Harris grew up in Indiana, and he graduated from Indiana University School of Medicine in 1973. After the completion of a general surgery intern year, he trained under the tutelage of Albert Rhoton Jr., MD at the University of Florida. Dr. Harris graduated from residency in 1978 after learning innumerable valuable lessons from Dr. Rhoton’s vast knowledge of neuroanatomy. Upon graduation, he worked in Florida (Fort Lauderdale), California (Bakersfield), and Wisconsin (Marshfield) until 1994, at which time he joined the faculty of Scott and White Hospital. He remained head of the Division of Neurosurgery until 2006, and he is still an active member of the faculty, now specializing in tumors of the brain and spinal cord.

The Division of Neurosurgery became an independent department during the 2006-2007 academic year, and Dr. Robert Buchanan was the first Chair of the Department of Neurosurgery at Scott and White Hospital. Following Dr. Buchanan’s departure in 2009, there were multiple interim chairs, including Gerhard Friehs, MD, Ibrahim El Nihum, MD, and David Garrett, MD. During that time, the Department of Neurosurgery was also staffed by Frank Harris, MD, Charles Wright, MD, Theodore Spinks, MD, and Vasilios Zerris, MD, MPH.

In 2014, after an extensive national search, Jason Huang, MD was named as Chair of the Department of Neurosurgery and the Plummer Family Endowed Chair of Neuroscience. Dr. Huang came to the United States in 1992 and attended Amherst College, graduating magna cum laude in 1994. After spending a year doing neuroscience research at Brigham & Women’s Hospital at Harvard Medical School, he enrolled at Johns Hopkins University School of Medicine and graduated in 1999. He then went to the University of Pennsylvania for his general surgery intern year and neurosurgery residency, training under M. Sean Grady, MD [3]. During his residency, he completed enfolded fellowships in Peripheral Nerve Surgery, Complex Spine, as well as Neurotrauma and Critical Care. He finished training in 2006 and began his career as an Assistant Professor of Neurosurgery at the University of Rochester Medical Center in Rochester, New York. He was promoted to Associate Professor of Neurosurgery in 2010. He served as an attending of Neurosurgery at both Strong Memorial Hospital and Highland Hospital and established a busy academic neurosurgery practice there. In 2008, Dr. Huang was deployed to Balad Theater Hospital in Iraq to aid in the United States efforts of Operation Iraqi Freedom. For his service, he received an Army Commendation Medal, and he was honorably discharged in 2012 with the rank of lieutenant colonel. As Chair of the Department of Neurosurgery, Dr. Huang has overseen significant growth, which now boasts 11 primary neurosurgical faculty members. He has also overseen the initiation of the residency program as Program Director, which now has a full complement of seven residents.

The residency training program at Baylor Scott & White Health

The application for a residency program was submitted during the 2014-2015 academic year and approval for the program was granted by the ACGME with the first submission on February 12, 2015. Jason Huang, MD has served as Program Director since that time with support from various Associate Program Directors (Table 1). T. Matthew Robinson, MD was welcomed as the first resident in 2015, and he is currently an attending Cerebrovascular Neurosurgeon at the University of New Mexico Health Sciences Center. The program has now graduated four residents, three of whom have gone on to complete subspecialty fellowships (cerebrovascular, endovascular, and stereotactic radiosurgery).

**Table 1:** Residency Leadership List of Program Directors, Associate Program Directors, and graduating Chief residents.

Year	Program Director	Associate Program Director	Graduating Chief Resident(s)
2014-2015	Jason Huang, MD	None	
2015-2016	Jason Huang, MD	None	
2016-2017	Jason Huang, MD	None	
2017-2018	Jason Huang, MD	David Garrett Jr., MD	
2018-2019	Jason Huang, MD	David Garrett Jr., MD	
2019-2020	Jason Huang MD	Rabia Qaiser, MD	T. Matthew Robinson, MD
2020-2021	Jason Huang, MD	Rabia Qaiser, MD	Sam Dayawansa, MD
2021-2022	Jason Huang, MD	J Kevin Cooper, MD	Kris Lyon, MD; Buqing Liang, MD
2022-2023	Jason Huang, MD	Awais Vance, MD	Jose Miguel Soto, MD

**Figure 3:**
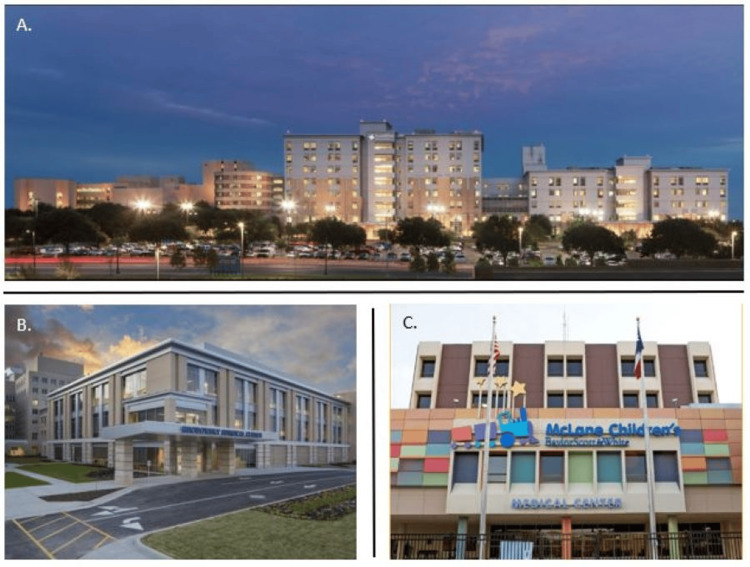
Baylor Scott & White Health A. Baylor Scott & White Health – Temple Campus. B. Grobowsky Surgical Center. C. McLane’s Children's Hospital. (Images were adopted from the Baylor Scott & White Health website, and permission has been granted to use them in this publication.)

The main campus for the program is Baylor Scott & White Memorial Hospital (Figure 3A), which is a Comprehensive Stroke Center certified by the Joint Commission, a Level 1 trauma center certified by the American College of Surgeons, and has been ranked as the top major teaching hospital in the United States [1]. There are 32 operating rooms at the Growbosky Surgical Center on the main campus (Figure 3B), including a biplane suite for endovascular neurosurgery. The residents also rotate at McLane’s Children’s Hospital, a Level II pediatric trauma center with five operating rooms (Figure 3C), and at Baylor Scott & White - Hillcrest in Waco, Texas. Subspecialty rotations include cerebrovascular, trauma, spine, pediatric neurosurgery, neuro-oncology, neuroradiology, functional neurosurgery, and neuro-intensive care. These rotations are supplemented by active participation in regularly scheduled conferences, including Case Conference, Journal Club, Neuroradiology Conference, Neuropathology (brain cutting), Tumor Board, Stroke/Large Vessel Occlusion Conference, faculty lectures, and cadaver labs.

**Figure 4:**
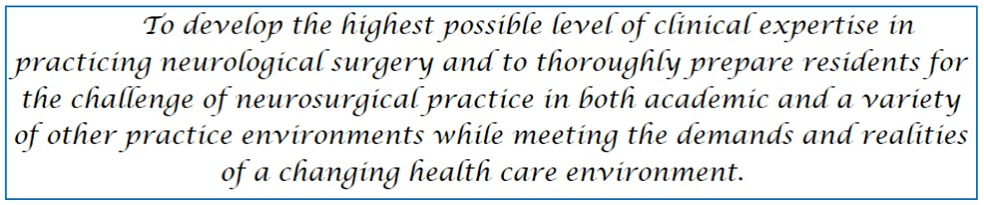
Educational Goal Educational goal of the neurosurgery residency training program at Baylor Scott & White Health/Texas A&M School of Medicine.

The educational goal of the neurosurgery residency program is highlighted in Figure 4. Residents are assigned a faculty mentor for each academic year, and they each meet with the Program Director every six months to formally evaluate their progress toward that goal as well as each of the ACGME core competencies for their specific level of training. Residents are also provided the opportunity to evaluate the faculty through anonymous online forms. A Resident Evaluation Committee, composed of each of the core faculty and the Program Director, meets yearly to evaluate the residents, and a written summary of the resident's evaluation is provided to them.

Academic productivity is actively supported and encouraged throughout the seven-year program. Residents are encouraged to undertake a clinical or basic science-level research project during their PGY-1 or PGY-2 year, and all residents meet on a monthly basis with the Associate Program Director to review academic productivity. The fifth year of training is designated as an elective/research year that residents have used for a wide variety of opportunities, including basic science research, enfolded fellowships, clinical research, and focused subspecialty training. Research activity is expected to result in abstract presentations at national neurosurgery meetings and manuscripts for peer-reviewed publication. During the most recent academic year, our residents averaged 3.5 publications per resident.

Current state and future directions of the Department of Neurosurgery

**Figure 5:**
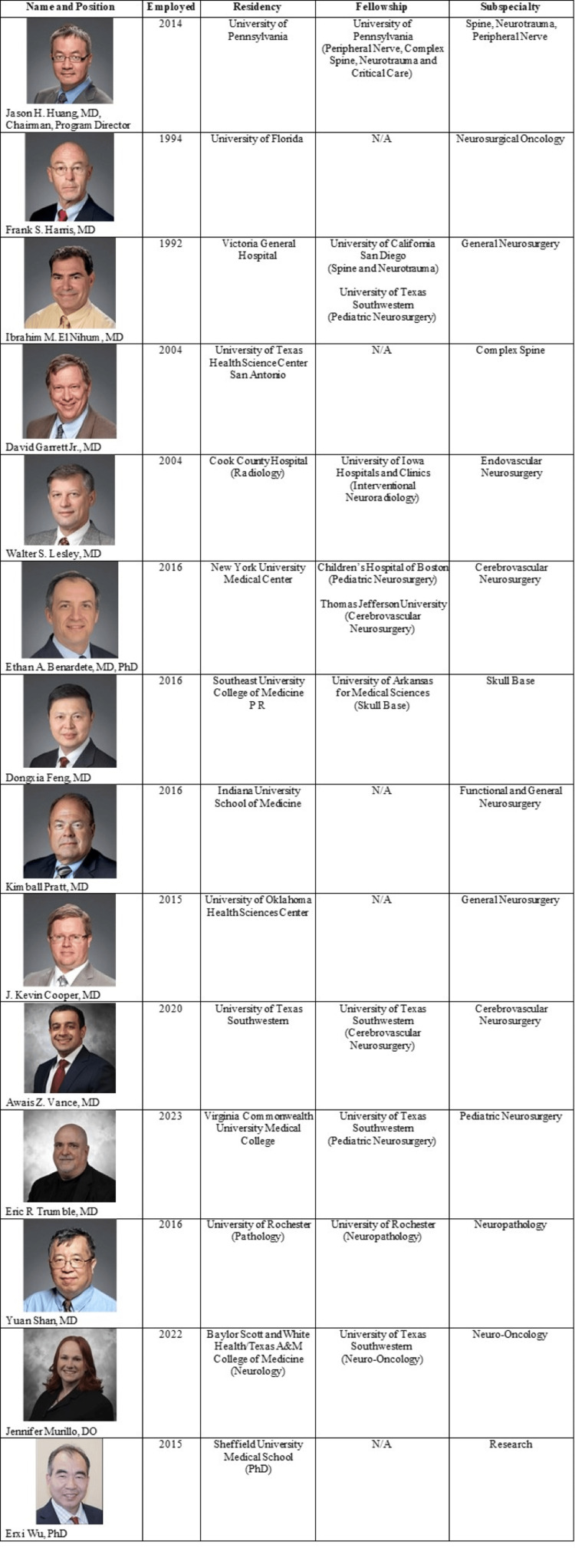
Department of Neurosurgery Faculty of the Department of Neurosurgery at Baylor Scott & White Health. (Images were adopted from the Baylor Scott & White Health website, and permission has been granted to use them in this publication.)

The Department of Neurosurgery has now grown to include 11 primary neurosurgery faculty (Figure 5), and other departments have added faculty to support the growing department. A new faculty member was hired in Neuro-oncology, and the Pathology Department is planning to hire an additional Neuropathologist. The institution also established a dedicated Neuroscience Intensive Care Unit in July 2022 to support increased patient volumes, and efforts are being made to keep it staffed exclusively by board-certified Neurocritical Care intensivists. Baylor College of Medicine recently partnered with Baylor Scott & White Health to establish a medical school in Temple, Texas, and the inaugural class of 40 medical students was welcomed in August 2023. Over the next four years, the campus will grow to 160 students, substantially increasing the opportunities for medical student involvement within the department.

The department's research capabilities have been significantly enhanced since Dr. Erxi Wu assumed the role of Vice Chair for Research in the Department of Neurosurgery in 2020. Dr. Wu has built upon the foundations he began laying upon arrival at our institution in 2015, and he has fostered a dynamic and productive research environment. Under his guidance and in collaboration with our residents and clinical faculty members, the research team has published several hundred peer-reviewed papers since 2015. These publications include impactful papers in prestigious journals such as *Nature Medicine*, *Nature Communications*, *Cancer Research*, *Brain*, *Journal of Neurosurgery*, *Neurosurgery*, *Journal of Neurotrauma*, *Journal of the American Chemical Society*, and *Alzheimer’s & Dementia*. The department’s research endeavors have not only elevated its academic profile but have also been instrumental in securing substantial external funding. This includes prestigious grants from the National Institutes of Health, which have been crucial in supporting the department's research endeavors. During their elective years, neurosurgery residents have the unique opportunity to engage in bench research for a year at the state-of-the-art laboratory facilities on the West Campus. Under the mentorship of Dr. Wu and other faculty members, residents are able to delve into groundbreaking translational research projects, equipping them with invaluable skills and knowledge that will serve them in their future careers in academic neurosurgery.

These improvements and areas of growth have allowed the program to continue to rise in national rankings. Most recently, it was ranked in the top 50% of neurosurgery training programs in the United States, and it is the third-ranked program of its size [4]. The main campus was re-certified in 2021 by the Joint Commission as the only Comprehensive Stroke Center in the Baylor Scott & White Health system, and as previously mentioned, it was ranked as the top major teaching hospital in the United States [1]. These positive recognitions to the hospital have significantly increased the ability to attract talented individuals, diversified the training environment, and enriched the teaching climate for neurosurgery residents.

The vision of the Department of Neurosurgery is to provide our patients with life-changing innovative treatment and care. We do this by training and educating the best neurosurgeons in the country, conducting cutting-edge clinical and translational research, and providing a constant, consistent, and rigorous continuing education curriculum to students, residents, staff, and physicians. As the population of Central Texas continues to grow [5], the Department of Neurosurgery will maintain its role as a full-service department, treating all diseases of the nervous system in both pediatric and adult patients, and it will grow in both size and academic productivity to meet the demands of the future.
